# The Book World of Medicine and Science

**Published:** 1895-09-28

**Authors:** 


### The Book World of Medicine and Science.

The Law and Chemistry of Food and Drugs. By Dr. H. M. Robinson and C. H. Cribb, B.Sc. Small 8vo., 500 pp. (London: F. J. Rebman, 1895.)

The publication of this book comes at a very opportune time, seeing that the subject of food adulteration has been discussed before a Select Committee of the House of Commons during the last session of Parliament. The Act of 1875, whatever be its faults, has undoubtedly been of incalculable service in the repression of food adulteration in this country, and would have served its purpose still better if in a few respects the legal aspect of the question had been modified so as to remove the difficulties which have arisen in the law courts through vendors taking the opportunity of making use of the various loopholes and legal quibbles which a careful study of the Act has shown to exist in it. It would seem from the interpretation of the present Act that it is not adulteration but fraud to sell margarine as butter, and that whilst it is neither adulteration nor fraud to sell a mixture of pure mustard and wholesome condiment as mustard, it is fraud and not adulteration to sell exhausted ginger as ginger. When a mixture of coffee and chicory is sold as coffee, and at the price of the best quality coffee, a double offence of adulteration and fraud is committed by the vendor. These points, and a great many more, are discussed in the book under review, and we are of the opinion that the combination of authors representing the legal and chemical aspect of the case has resulted in a book which must find its place on the shelves of all concerned in the working of the present Act and other statutes dealing with the laws relating to the adulteration and unsoundness of food and drugs.

Although the evidence given before the Select Committee shows that the problem of amending these Acts is fraught with considerable difficulty, it is to be hoped that in the forthcoming session a new Act will be passed which will, amongst other things, simplify the vexed "warranty" question, and place the Somerset House reference in its proper position, which shall be alike fair to the vendor and the public analyst representing the local authority. The whole subject involves ethical and economical issues of considerable importance, and it is high time that England, who at one time led the nations in her enactments for the supression of adulteration, should once more place her laws in a condition which will make them at least equivalent in severity on unjust trading to those which obtain in the Colonies and on the Continent. In the very excellent article on antiseptics as adulterants we find no mention of formalin as a common preservative of milk and other perishable articles, and must take exception to Mr. Cribb's statement that the hydrocarbons are fats containing carbon, hydrogen, and oxygen, and that they are necessary for the production of heat and force. The author's chemistry, physics, and literary ability are evidently weak just there. We hope that a new Act will soon be passed, and so call for a new edition of this eminently practical and useful book.

